# Prognostic value of baseline neutrophil/lymphocyte ratio in HER2-positive metastatic breast cancer: exploratory analysis of data from the CLEOPATRA trial

**DOI:** 10.1186/s13058-023-01761-x

**Published:** 2024-01-11

**Authors:** Nianhua Ding, Jian Pang, Xuan Liu, Xiongbin He, Wei Zhou, Haiqing Xie, Jianqi Feng, Guo Wang, Jie Tang, Jing Cao, Liying He, Yingjian He, Shouman Wang, Zhi Xiao

**Affiliations:** 1 Department of Clinical Laboratory, The Affiliated Changsha Hospital of Xiangya School of Medicine, Central South University, Changsha, China; 2 Department of General Surgery, The Second Xiangya Hospital, Central South University, Changsha, People’s Republic of China; 3 Department of General Surgery, The First People’s Hospital of Xiangtan, Xiangtan, People’s Republic of China; 4 Department of Breast and Thyroid Surgery, The First People’s Hospital of Chenzhou, Chenzhou, People’s Republic of China; 5 Department of Breast Surgery, The Affiliated Zhuzhou Hospital of Xiangya School of Medicine, Zhuzhou, People’s Republic of China; 6 Department of Breast and Thyroid Surgery, The Third People’s Hospital of Chenzhou, Chenzhou, People’s Republic of China; 7 Department of Breast and Thyroid Surgery, The First People’s Hospital of Huaihua, Huaihua, People’s Republic of China; 8 Department of Clinical Pharmacology, Xiangya Hospital, Central South University, Changsha, People’s Republic of China; 9 Department of Breast SurgeryXiangya Hospital, Central South University, Changsha, People’s Republic of China; 10 Key Laboratory of Carcinogenesis and Translational Research (Ministry of Education/Beijing), Breast Center, Peking University Cancer Hospital and Institute, Beijing, People’s Republic of China; 11 Department of General Surgery, Xiangya Hospital, Central South University, Changsha, People’s Republic of China; 12 Department of Breast Surgery, General Surgery, Xiangya Hospital, Central South University, 87 Xiangya Road, Changsha, 410008 Hunan People’s Republic of China; 13 Clinical Research Center for Breast Cancer in Hunan Province, Changsha, People’s Republic of China; 14 Multidisciplinary Breast Cancer Center, Xiangya Hospital, Central South University, Changsha, People’s Republic of China

**Keywords:** Neutrophil/lymphocyte ratio, HER2-positive, Metastatic breast cancer, Trastuzumab/pertuzumab, Prognostic value

## Abstract

**Purpose:**

This study aimed to evaluate the prognostic role of the baseline neutrophil/lymphocyte ratio (NLR) in HER2-positive metastatic breast cancer (MBC) patients treated with trastuzumab/pertuzumab.

**Experimental design:**

Data from 780 patients from the CLEOPATRA trial and 248 local patients were collected. Patients were divided into the low and high NLR subgroups by the NLR cutoff value. Propensity score matching (PSM) and inverse probability of treatment weighting (IPTW) methods were used to control bias. Associations between the NLR and progression-free survival (PFS) and overall survival (OS) were analyzed.

**Results:**

The baseline characteristics of the subgroups were well balanced after PSM and IPTW. A low baseline NLR was associated with better PFS and OS in the trastuzumab and docetaxel (TH) group in the unadjusted, PSM and IPTW models. After IPTW, a low NLR, versus a high NLR, was associated with improved PFS (HR 1.35, 95% CI 1.07–1.70, *P* = 0.012) and OS (HR 1.47, 95% CI 1.12–1.94, *P* = 0.006) in the TH group. In patients undergoing treatment with trastuzumab and pertuzumab and docetaxel (THP), a low baseline NLR was also correlated with better PFS but not OS across the three models. After IPTW, a low NLR was associated with better PFS (HR 1.52, 95% CI 1.20–1.93, *P* = 0.001) than a high NLR in the THP group. Multivariate analyses showed that a low baseline NLR was a predictor for PFS and OS in the TH group and for PFS in the THP group in all three models. In the real-world setting, a low baseline NLR was a predictor of better PFS among patients treated with docetaxel plus trastuzumab without or with pertuzumab in the multivariate model (*P* = 0.015 and 0.008, respectively).

**Conclusions:**

A low baseline NLR is associated with better survival outcomes among HER2-positive MBC patients receiving docetaxel plus trastuzumab/pertuzumab as first-line therapy.

**Supplementary Information:**

The online version contains supplementary material available at 10.1186/s13058-023-01761-x.

## Introduction

Breast cancer (BC) has the highest incidence and is the leading cause of cancer-related mortality for females in China [1, 2]. HER2-positive BC accounts for 15–20% of all invasive BC cases and is characterized by aggressive tumor cell proliferation and a poor prognosis. Trastuzumab and pertuzumab are effective therapies for HER2 overexpressing BC [3, 4]. The antitumor mechanisms of trastuzumab and pertuzumab include preventing dimer formation, inhibiting kinases of downstream signaling pathways, and inducing antibody-dependent cell-mediated cytotoxicity (ADCC) [5–7]. Meanwhile, resistance to trastuzumab and pertuzumab exists, and the resistance mechanism is not yet understood [8]. The identification of factors predicting sensitivity to trastuzumab and pertuzumab among HER2-positive BC patients is urgently needed to differentiate patients who will benefit more from anti-HER2 treatment from those who will benefit less.

The systemic inflammatory status of the host and the inflammatory response in the tumor microenvironment play critical roles in cancer initiation, invasion, and metastasis [9, 10]. The NLR has been recognized as an indicator of the host’s systemic inflammatory status and is associated with prognosis in many solid tumors [11, 12]. Many studies have shown that a higher NLR is correlated with worse survival in triple-negative BC; however, the relationship remains controversial in HER2-positive BC [13–16].

In our previous study, we showed that a lower baseline NLR was correlated with better survival among HER2-positive BC patients receiving adjuvant trastuzumab therapy [16]. The current study aimed to explore whether the baseline NLR holds prognostic value among HER2-positive metastatic breast cancer (MBC) patients receiving trastuzumab/pertuzumab as the first-line therapy. In this study, we reanalyzed the data of 790 HER2-positive MBC patients from the CLEOPATRA trial (NCT00567190). Furthermore, data of 248 HER2-positive MBC patients receiving trastuzumab/pertuzumab from six local hospitals were evaluated to confirm the prognostic value of the NLR.

## Patients and methods

### Study design and data source

**Fig. 1:**
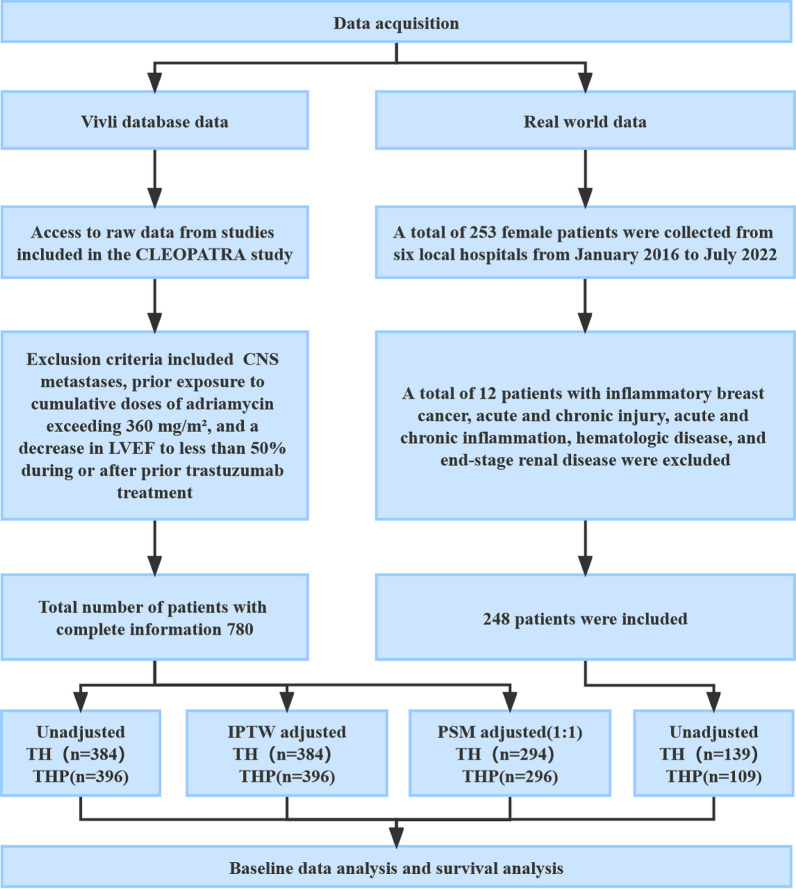
The flowchart of our study

Details of the CLEOPATRA study have been published previously [17]. Briefly, the CLEOPATRA trial was a double-blind, randomized, placebo-controlled phase 3 clinical trial that enrolled 808 patients at 204 centers in 25 countries between February 2008 and July 2010 to compare the efficacy and safety of trastuzumab and docetaxel with placebo or pertuzumab in patients with HER2-positive MBC. Of all these patients, 406 were randomly assigned to receive placebo plus trastuzumab plus docetaxel (TH group), and 402 were randomly assigned to receive pertuzumab plus trastuzumab plus docetaxel (THP group). The main inclusion for the CLEOPATRA study criteria were as follows: (1) metastatic HER2-positive breast cancer; (2) age of 18 years or older; (3) Eastern Collaborative Oncology Group (ECOG) performance status of 0 or 1; (4) a left ventricular ejection fraction (LVEF) of at least 50%; (5) not receiving chemotherapy or biologic therapy for metastatic disease; and (6) receiving up to one hormone therapy for metastatic disease, but not concurrent hormone therapy before disease progression. The exclusion criteria were as follows: (1) central nervous system metastases from breast cancer; (2) cumulative doses of doxorubicin exceeding 360 mg/m2 from prior therapy; and (3) a decrease in LVEF below 50% during or after prior trastuzumab therapy. In this study, clinical data from the CLEOPATRA trial were obtained from VIVLI (https://vivli.org/) for 780 patients with complete follow-up information, including 384 patients in the TH group and 396 in the THP group. The study flowchart of the CLEOPATRA trial and real-world research is shown in Fig. 1.

### Real-world research

To validate the findings from the CLEOPATRA trial, we collected data from HER2-positive MBC patients who had received first-line therapy with pertuzumab/trastuzumab plus docetaxel for metastatic disease. A total of 248 female patients from six local hospitals were enrolled from January 2016 to July 2022; each of these hospitals performed at least 300 breast cancer operations per year. Medical records were reviewed to collect and organize the patients’ age, medical history, laboratory test results, and hormone receptor status. HER2 positivity was defined as an immunohistochemical staining result of 3 + or 2 +, but FISH positivity was defined as a HER2/CEP17 ratio > 2.2. Patients with inflammatory breast cancer, acute or chronic injury, acute or chronic inflammation, a hematological disorder, or end-stage renal disease were excluded.

This study was approved by the Institutional Review Board of Xiangya Hospital (202002021) and conducted under the guidance of the Declaration of Helsinki. All participants provided written informed consent to participate the study.

### NLR cutoff value

The NLR was calculated by dividing the absolute number of neutrophils by the absolute number of lymphocytes. In the TH group, a median NLR of 2.552 was adopted as the cutoff value, and the patients were divided into the low NLR subgroup (192 patients with an NLR < 2.552) and the high NLR subgroup (192 patients with an NLR ≥ 2.552), as done previously [16]. In the THP group, a median NLR of 2.479 was adopted as the cutoff value, and the patients were divided into the low NLR subgroup (197 cases with an NLR < 2.479) and the high NLR subgroup (199 cases with an NLR ≥ 2.479).

In the real-world research of our local patients, the median NLR of 2.571 was used as the cutoff value for patients receiving treatment with trastuzumab plus docetaxel (rTH group), and the median NLR of 2.80 was used for patients receiving treatment with pertuzumab plus trastuzumab plus docetaxel (rTHP group).

### Statistical analysis

Patient demographic characteristics and baseline characteristics are summarized using descriptive statistics or columns. Differences in clinicopathological characteristics between the high and low NLR subgroups were assessed using chi-square tests or Fisher's exact test.

Progression-free survival (PFS) and overall survival (OS) were defined as in the CLEOPATRA trial and taken as the endpoints in this study. To account for selection bias and potential cofounding factors between the low and high NLR subgroups, propensity scores were used to control for differences in baseline characteristics between patients in the TH and THP groups. Two adjustment approaches based on propensity scores were used, propensity score matching (PSM) and inverse probability of treatment weighting (IPTW). The PSM approach was used to estimate the NLR effect for specific groups of patients after one-to-one matching, which might sacrifice data from the whole cohort of patients. The IPTW approach weighted each patient by the inverse probability of being in the low versus high subgroup. A propensity score for each patient was calculated as the predicted probability from multivariable logistic regression that included major confounding factors associated with survival: age, disease type at screening, ECOG performance status, hormone-receptor status and previous systemic therapy.

Survival curves were constructed using the Kaplan‒Meier (KM) method and log-rank test. Cox proportional risk regression analysis was used to estimate the hazard ratio (HR) for risk factors (95% confidence interval [CI]), and the results of Cox proportional risk regression analysis were visualized using forest plots. R Studio (R version 4.1.3) was used for data analyses and graphical plotting. All statistical analyses were considered statistically significant (two-sided *P* < 0.05).

## Results

### Patients' clinical baseline information from the CLEOPATRA trial

Before adjustment, in the TH group, there were more patients with an ECOG performance status ≥ 1 in the high NLR subgroup than in the low NLR subgroup (47.4% vs. 32.3%, *P* = 0.003). Other characteristics, such as age, disease type at screening, hormone-receptor status and previous neoadjuvant or adjuvant systemic therapy, were not significantly different between subgroups (Table 1). In the THP group, there was no significant difference between the high and low NLR subgroups in any of these characteristics (Table 1).

**Table 1:** Demographic and disease characteristics before and after IPTW

Characteristics	TH group (*n *= 384)				THP group (*n *= 396)			
	Low	High	*P* value	PS weighted *P*	Low	High	*P* value	PS weighted *P*
*Age, years-no. (%)*								
< 55	167 (87.0)	153 (79.7)	0.055	0.906	111 (56.3)	93 (46.7)	0.056	0.984
≥ 55	25 (13.0)	39 (20.3)			86 (43.7)	106 (53.3)		
*Disease type at screening-no. (%)*								
Nonvisceral	50 (26.0)	36 (18.8)	0.087	0.934	47 (23.9)	36 (18.1)	0.159	0.992
Visceral	142 (74.0)	156 (81.2)			150 (76.1)	163 (81.9)		
*ECOG performance status-no. (%)*								
0	130 (67.7)	101 (52.6)	**0.003**	0.853	140 (71.1)	131 (65.8)	0.262	0.991
≥ 1	62 (32.3)	91 (47.4)			57 (28.9)	68 (34.2)		
*Hormone-receptor status-no. (%)*								
Positive	95 (49.5)	90 (46.9)	0.069	0.977	99 (50.3)	109 (54.8)	0.420	0.697
Negative	88 (45.8)	100 (52.1)			97 (49.2)	90 (45.2)		
Unknown	9 (4.7)	2 (1.0)			1 (0.5)	0 (0)		
*Previous neoadjuvant or adjuvant systemic therapy-no. (%)*								
No	99 (51.6)	105 (54.7)	0.539	0.927	112 (56.9)	105 (52.8)	0.414	0.986
Yes	93 (48.4)	87 (45.3)			85 (43.1)	94 (47.2)		

Bold indicates *P* < 0.05

*IPTW* Inverse probability of treatment weighting, *TH group* Trastuzumab plus docetaxel group, *THP group* Pertuzumab plus trastuzumab plus docetaxel

After adjustment with PSM or IPTW, the baseline characteristics were balanced between the high and low NLR subgroups in both the TH and THP groups, as shown in Table 1 and Additional file 1: Tables S1, S2, S3 and S4.

### Patients’ clinical baseline information from real-world research

In the real-world study, in the TH group with 139 patients, more patients had a positive hormone receptor status in the high NLR subgroup than in the low NLR subgroup (84.7% vs. 62.7%, *P* = 0.003). Other characteristics, such as age, disease type at screening, ECOG performance status and trastuzumab treatment in the neoadjuvant setting, were not significantly different between subgroups (Additional file 1: Table S5). In the THP group with 109 patients, more patients had a negative hormone receptor status in the high NLR subgroup than in the low NLR subgroup (64.8% vs. 21.8%, *P* < 0.001). Other characteristics, such as age, disease type at screening and ECOG performance status, were not significantly different between subgroups (Additional file 1: Table S5).

### Survival analyses of patient from the CLEOPATRA trial

**Fig. 2:**
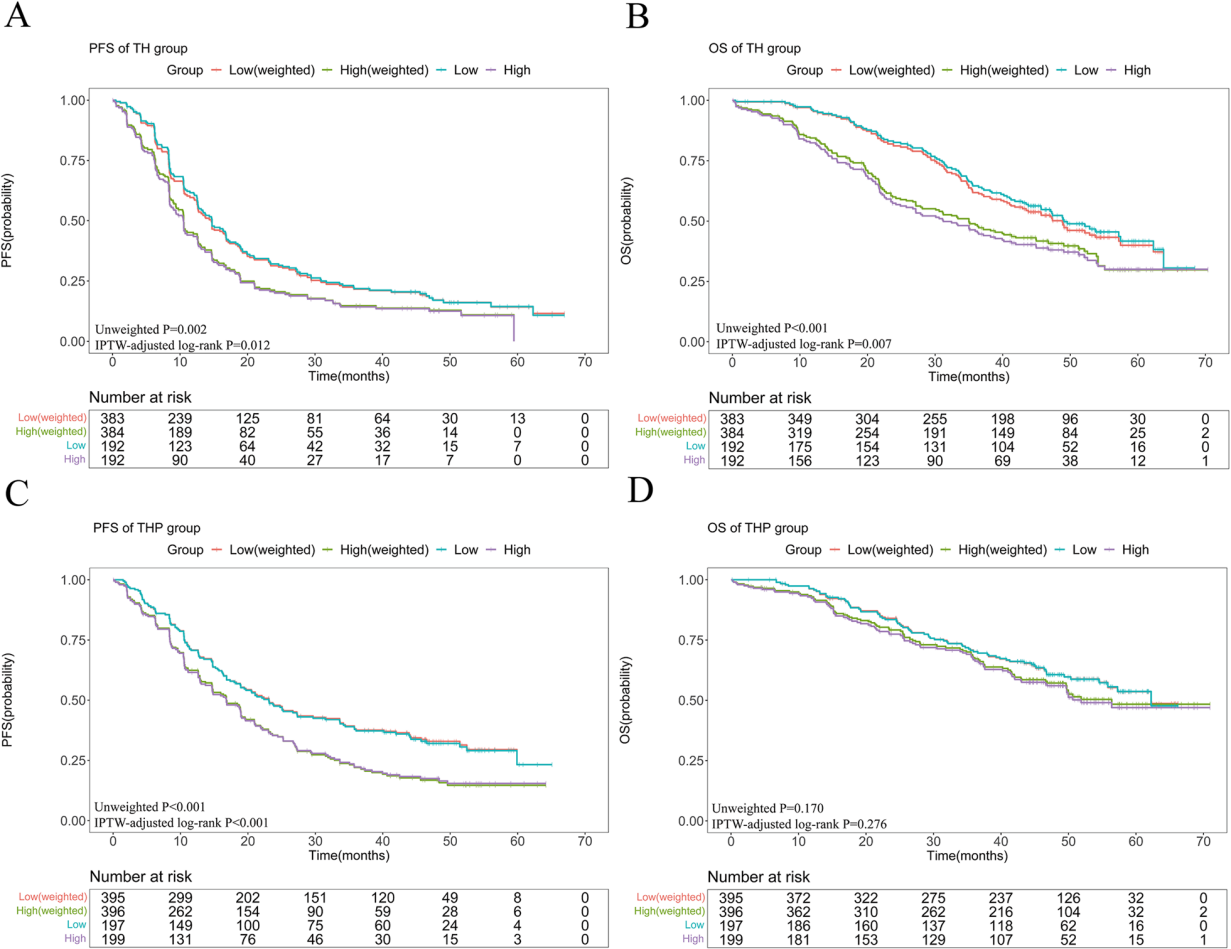
The KM curves of **A** PFS and **B** OS before and after IPTW analyses according to the low or high NLR in TH group. The KM curves of **C** PFS and **D** OS before and after IPTW analyses according to the low or high NLR in THP group. Abbreviations: KM, Kaplan–Meier; PFS, progression-free survival; OS, overall survival; IPTW, inverse probability of treatment weighting; TH group, trastuzumab plus docetaxel group; THP group, pertuzumab plus trastuzumab plus docetaxel

In the raw data of the TH group, the median PFS was 14.7 months (95% CI 12.0–17.5) in the low NLR subgroup versus 10.5 months (95% CI 9.1–11.9) in the high NLR subgroup (HR 1.42, 95% CI 1.13–1.78,* P* = 0.002); the median OS was 49.1 months (95% CI 41.5–56.6) in the low NLR subgroup versus 31.2 months (95% CI 24.4–38.0) in the high NLR subgroup (HR 1.66, 95% CI 1.26–2.19, *P* < 0.001). In the raw data of the THP group, the median PFS was 23.1 months (95% CI 17.8–28.4) in the low NLR subgroup versus 16.8 months (95% CI 13.8–19.8) in the high NLR subgroup, with a statistically significant difference (HR 1.50, 95% CI 1.18–1.90, *P* < 0.001); there was a trend showing that patients in the low NLR subgroup had better OS than those in the high NLR subgroup (*P* = 0.170) (Fig. 2).

After IPTW, a low NLR was still associated with significantly improved survival in the TH group (HR 1.35, 95% CI 1.07–1.70, *P* = 0.012 for PFS; HR 1.47, 95% CI 1.12–1.94, *P* = 0.006 for OS). In the THP group, similar results as those before IPTW were obtained, which showed that a low NLR was associated with significantly better PFS (HR 1.52, 95% CI 1.20–1.93, *P* = 0.001). However, the difference in OS was not statistically significant (HR 1.19, 95% CI 0.87–1.62, *P* = 0.272) (Fig. 2).

After PSM, we also obtained similar results. In the TH group, patients in the low NLR subgroup had significantly better PFS and OS (*P* = 0.030, *P* = 0.007, respectively) (Additional file 1: Fig. S1). In the THP group, a low NLR was associated with better PFS (*P* = 0.003) but not OS (*P* = 0.490) (Additional file 1: Fig. S1).

### Factors influencing prognosis of patients in the CLEOPATRA trial

**Fig. 3:**
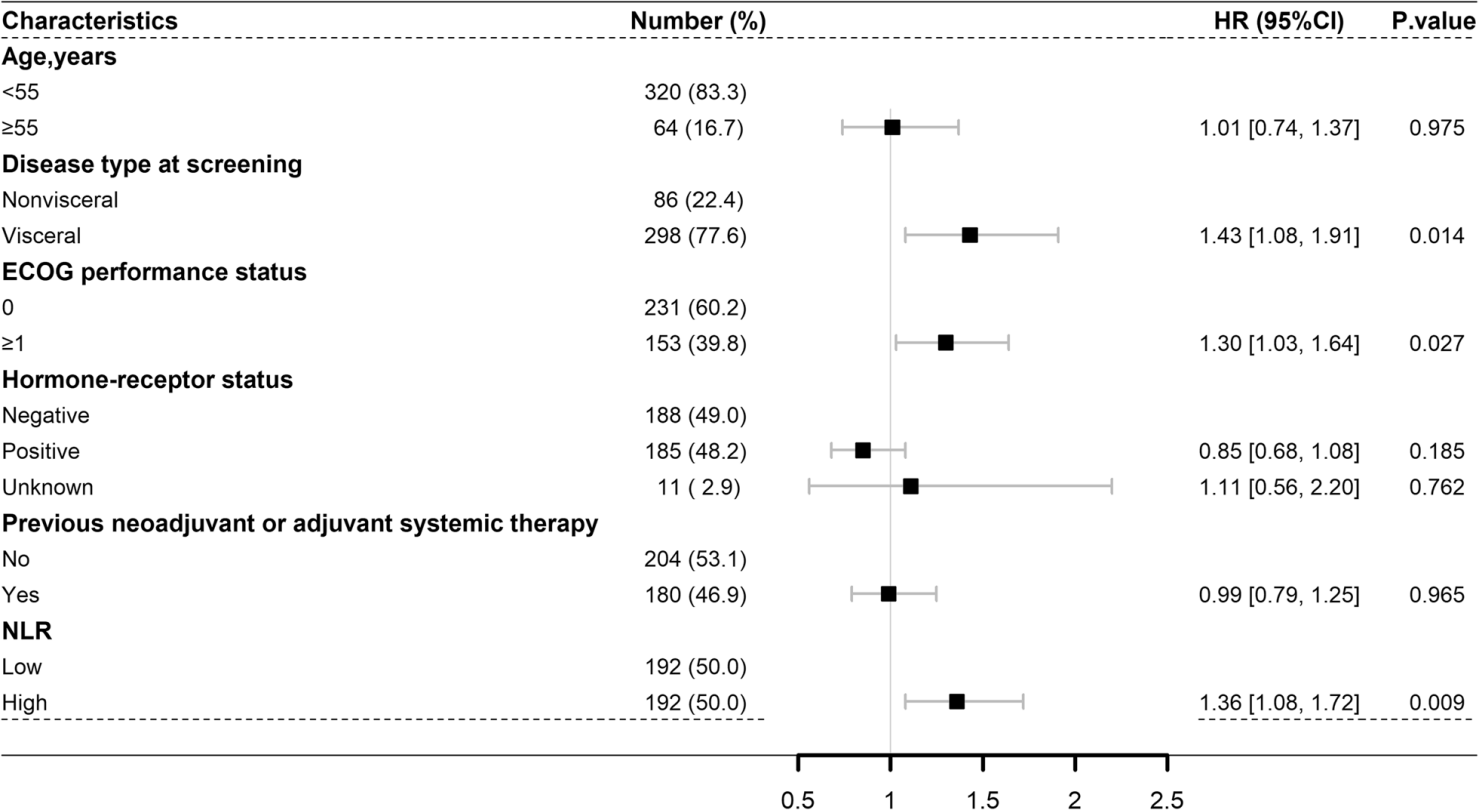
Forest plots showed independent influences on PFS in the TH group by multivariate analysis. Abbreviations: HR, hazard ratio; 95% CI, 95% confidence interval; NLR, neutrophil to lymphocyte ratio; PFS, progression-free survival; TH group, trastuzumab plus docetaxel group

Before IPTW adjustment, multivariate analysis revealed that the disease type (visceral vs. nonvisceral: HR 1.43, 95% CI 1.08–1.91,* P* = 0.014), ECOG status (≥ 1 vs. 0: HR 1.30 95% CI 1.03–1.64, *P* = 0.027), and NLR (high vs. low: HR 1.36, 95% CI 1.08–1.72, *P* = 0.009) were independent predictors of PFS in the TH group (Fig. 3). The disease type, ECOG performance and NLR were also significantly associated with OS in the TH group (Additional file 1: Fig. S2). In the THP group, the data showed that the NLR was also an independent predictor of PFS (high vs. low, HR 1.53, 95% CI 1.21–1.95, *P* < 0.001) (Additional file 1: Fig. S3) but not OS (Additional file 1: Fig. S4).

After IPTW, the clinical characteristics in the low and high NLR subgroups were well balanced (Additional file 1: Tables S3 and S4). In the TH group, the IPTW-adjusted analyses showed that the NLR was still an independent predictor of PFS (high versus low: HR 1.38, 95% CI 1.09–1.74, *P* = 0.007) (Table 2) and OS (high versus low: HR 1.60, 95% CI 1.21–2.13, *P* = 0.001) (Additional file 1: Table S6). In the THP group, the IPTW-adjusted analyses demonstrated that the NLR was an independent predictor of PFS (high versus low: HR 1.54, 95% CI 1.21–1.95,* P* < 0.001) (Additional file 1: Table S7) but not OS (Additional file 1: Table S8).

**Table 2:** IPTW-adjusted univariate and multivariate analyses of the relationship between PFS and clinical factors in TH group

Factor	Univariate analysis			Multivariate analysis		
	HR	95%CI	*P* value	HR	95%CI	*P* value
Age, years (≥ 55 vs. < 55)	1.030	0.74–1.45	0.853	0.980	0.69–1.39	0.905
Disease type at screening (visceral vs. nonvisceral)	1.440	1.10–1.88	**0.008**	1.420	1.08–1.86	**0.011**
ECOG performance status (≥ 1 vs. 0)	1.290	1.02–1.65	**0.037**	1.260	0.98–1.61	0.074
*Hormone-receptor status*						
Negative vs. positive	1.070	0.85–1.36	0.553	1.130	0.89–1.44	0.320
Unknown vs. positive	1.310	0.92–1.87	0.136	1.440	1.07–1.93	**0.016**
Previous neoadjuvant or adjuvant systemic therapy (YES vs. no)	1.020	0.81–1.29	0.854	1.010	0.79–1.28	0.956
NLR (High vs. low)	1.350	1.07–1.70	**0.012**	1.380	1.09–1.74	**0.007**

Bold indicates *P* < 0.05

*HR* Hazard ratio, *95% CI* 95% Confidence interval, *NLR* Neutrophil to lymphocyte ratio, *IPTW* Inverse probability of treatment weighting, *TH group* Trastuzumab plus docetaxel group, *PFS* Progress-free survival

After PSM, the data were balanced among subgroups (Additional file 1: Tables S1 and S2). In the TH group, multivariate analyses demonstrated that the NLR was still an independent predictor of PFS and OS (Additional file 1: Table S9). In the THP group, PSM-adjustment analyses showed that the NLR was an independent predictor of PFS but not OS (Additional file 1: Table S10).

### Real-world research

Data of subgroups from our local hospitals were balanced for all characteristics except the hormone receptor status (Additional file 1: Table S5). The median PFS was 15 months for all patients from our local hospitals. Multivariate analyses revealed that the NLR was an independent predictor of PFS in the rTH group (high versus low: HR 1.67, 95% CI 1.10–2.53, *P* = 0.015) and in the rTHP group (high versus low: HR 2.04, 95% CI 1.20–3.46, *P* = 0.008) (Additional file 1: Table S11).

## Discussion

In this study, we showed that a low baseline NLR was significantly associated with better PFS and OS among HER2-positive MBC patients receiving trastuzumab plus docetaxel therapy by reanalyzing data from the CLEOPATRA trial. A low baseline NLR was significantly associated with improved PFS, but not OS, among HER2-positive MBC patients receiving pertuzumab plus trastuzumab plus docetaxel therapy in the CLEOPATRA trial. Similar PFS findings were further confirmed by a retrospective study of local data of HER2-positive MBC patients receiving pertuzumab/trastuzumab plus docetaxel treatment as the first-line therapy.

The NLR is an inexpensive and readily available marker of the systemic inflammatory response and tumor prognosis. A high NLR has been consistently recognized as a poor prognostic factor among triple-negative BC patients in meta-analyses, but not among HER2-positive BC patients [18, 19]. A study of 187 early HER2-positive BC patients who received adjuvant trastuzumab suggested that a low baseline NLR might be correlated with improved disease-free survival (DFS) outcome, but the difference was not significant [13]. Another retrospective study, involving 40% (43/107) of early HER2-positive BC patients receiving adjuvant trastuzumab determined that a high NLR was associated with notably lower survival rates only in the subgroup of HER2-positive patients not treated with trastuzumab, but not in the subgroup of patients who were treated with trastuzumab [14]. Conversely, our previous study of a total of 843 early HER2-positive BC patients (588 of whom received adjuvant trastuzumab for 1 year) demonstrated a significant association between a low baseline NLR and improved DFS among patients receiving trastuzumab but not in HER2-positive BC patients not receiving trastuzumab [16]. There are several reasons for these contradictory results. First, each study used a different method to determine the NLR cutoff point. Second, treatment regimens differed, particularly with regard to chemotherapy regimens. Last, the timing and duration of trastuzumab treatment varied across studies. Given that our previous study had the largest sample size and the most homogenous patient characteristics compared to the other two studies, the evidence supporting our finding that a low baseline NLR was associated with better DFS in HER2-positive patients receiving adjuvant trastuzumab treatment should be the most compelling.

The aforementioned studies only included HER2-positive early BC patients receiving adjuvant trastuzumab. Therefore, in this study, we sought to explore the predictive value of the NLR for PFS and OS in HER2-positive MBC patients. We reanalyzed data from the CLEOPATRA trial. In the TH group, HER2-positive MBC patients were divided into two subgroups based on the median NLR, as in our previous study of HER2-positive early BC [16]. The data of the subgroups in the TH group were even except for the ECOG status before adjustment and were excellently balanced after adjustment by PSM and IPTW. The Cox analysis of the TH group showed that the NLR consistently functioned as an independent predictive factor of PFS and OS among HER2-positive MBC patients receiving trastuzumab therapy, regardless of whether the data were adjusted. In the analysis of our local HER2-positive MBC patients receiving trastuzumab as first-line therapy, the same results were observed: patients with a low NLR exhibited notably extended PFS compared with those with a high NLR, which was consistent with the results reported by Shao et al. [20]. This indicates that HER2-positive MBC patients with a low baseline NLR might derive more benefit from trastuzumab than those with high baseline NLR, which could be attributed to trastuzumab-induced ADCC [21], as we have previously hypothesized, given that its efficacy might be intertwined with the host immune status. ADCC might be more efficiently triggered by trastuzumab in hosts with a healthy immune status (low NLR), and less so in hosts with a suppressed immune system (high NLR). In summary, we deduced that trastuzumab could elicit a stronger antitumor immune response through ADCC in patients with a low baseline NLR than in those with a high baseline NLR. This contradicts the hypothesis that trastuzumab can overcome the poor prognostic impact of a high NLR [14]. While we acknowledge that trastuzumab could modulate systemic inflammation and activate ADCC, we assert that this modulation is dependent on the immune status of the host, without which trastuzumab could not stimulate the immune system to attack tumor cells.

In the THP group, we also divided HER2-positive MBC patients into two subgroups according to the median NLR, and the subgroup data were even. The findings revealed that a low NLR was associated with improved PFS and OS in HER2-positive MBC patients receiving pertuzumab plus trastuzumab plus docetaxel (*P* < 0.001 and 0.170, respectively). These results were further corroborated after data adjustment through PSM or IPTW. In the analysis of our local HER2-positive MBC patients receiving the same treatment, patients with a lower NLR exhibited longer PFS, as indicated in this study. This seemed to be opposite to the results reported by Ligorio et al. and Araki et al.*,* who found that the pan-immune-inflammatory value or baseline absolute lymphocyte count, but not the NLR, was significantly associated with the prognosis of HER2-positive advanced BC patients treated with trastuzumab plus pertuzumab [22, 23]. Explanations for the inconsistent results include selection bias and the small sample size of these two retrospective studies (57 and 51 patients, respectively). Interestingly, we noticed a trend toward better survival associated with a low NLR in both studies mentioned above (*P* = 0.1 and 0.0548, respectively), aligning with our findings in this study. In terms of mechanism, one study showed that the combination of trastuzumab and pertuzumab amplified the ADCC reaction and induced more intensive growth inhibition of tumor cells [24]. Nevertheless, in our study, there was no statistically significant association between the NLR and OS in the THP group. This might be explained by the saturation of ADCC in vivo, which is influenced by various factors, such as the NK cell count and FcγRIII receptor affinity [24].

In the real-world study of our local patients, there was a difference in the distribution of patients’ hormone receptor status between the rTH and rTHP groups. This was attributed to the retrospective nature of the study of our local patients. However, this would not influence the selection of docetaxel plus trastuzumab/pertuzumab as the first-line treatment for HER2-positive MBC. Additionally, endocrine therapies were not included in the first-line treatments. In our opinion, this imbalance in hormone receptor status had no effect on PFS in this study.

An advantage of this study is that the analyzed data were extracted from prospective controlled database, which minimized missing data and patients lost to follow-up. To the best of our knowledge, this is the first study utilizing prospective data to assess the prognostic effect of the NLR on the survival of HER2-positive MBC patients. Another advantage is that we took steps to mitigate potential biases. Some baseline characteristics, such as the ECOG status, of patients in the subgroup were uneven; we employed two different adjustment approaches to reaffirm the value of the NLR with less confounding bias or imbalance in covariates and thus potentially supplied an estimation of the prognostic effect akin to that in randomized trials [25]. In our study, regardless of whether the PSM or IPTW approach was applied, the conclusions were the same and were consistent with the results obtained from the unadjusted data. In short, our results remained consistent and convincing.

There are some limitations to this study. First, the approach to define the cutoff point for the NLR differed among various studies; even applying the same approach in this study, the cutoff point for the NLR was different in the CLEOPATRA trial and the real-world research, but was approximately 2.5. However, we have reanalyzed the data using the median value of the NLR of all CLEOPATRA trial patients, which is 2.509, and the results from the reanalysis are in line with the findings of our current analysis. Second, the data of our local HER2-positive MBC patients were collected retrospectively, making bias and imbalance unavoidable. The small sample size was also an important issue. Reanalyses of data from classical clinical trials and larger randomized studies are needed for further exploration of the relationship between systemic inflammatory/immune markers and trastuzumab/pertuzumab-induced ADCC.

## Conclusion

This study showed that a low baseline NLR was associated with better survival outcomes in HER2-positive MBC patients receiving first-line therapy with trastuzumab plus docetaxel or a combination of trastuzumab, pertuzumab, and docetaxel. The baseline NLR might serve as a valuable factor to distinguish between HER2-positive MBC patients who would benefit more from these treatments and those who would benefit less. Reanalyses of data from classical clinical trials and large randomized studies are needed to verify the prognostic role of the baseline NLR in HER2-positive MBC patients treated with trastuzumab/pertuzumab.

### Supplementary Information

**Additional file 1: Table S1.** Baseline information of patients in the TH group before and after PSM. **Table S2.** Baseline information of patients in the THP group before and after PSM. **Table S3.** Characteristics of the TH group patients before and after IPTW. **Table S4.** Characteristics of the THP group patients before and after IPTW. **Table S5.** Demographic and disease characteristics of local patients. **Table S6.** IPTW-adjusted univariate and multivariate analyses of the relationship between OS and clinical factors of TH group. **Table S7.** IPTW-adjusted univariate and multivariate analyses of the relationship between PFS and clinical factors of THP group. **Table S8.** IPTW-adjusted univariate and multivariate analyses of the relationship between OS and clinical factors of THP group. **Table S9.** PSM-adjusted univariate and multivariate analyses of the relationship between PFS or OS and clinical factors of TH group. **Table S10. **PSM-adjusted univariate and multivariate analyses of the relationship between PFS or OS and clinical factors of THP group. **Table S11.** Univariate and multivariate analyses of the relationship between PFS and clinical factors of local patients. **Fig. S1.** After PSM, the KM curves of **A** PFS and **B** OS according to the low or high NLR in TH group. After PSM, the KM curves of **C** PFS and **D** OS according to the low or high NLR in THP group. Abbreviations: KM, Kaplan-Meier; PSM, propensity score matching; PFS, progression-free survival; OS, overall survival; TH group, trastuzumab plus docetaxel group; THP group, pertuzumab plus trastuzumab plus docetaxel. **Fig. S2.** Forest plots showed independent influences on OS in the TH group by multivariate analysis. Abbreviations: HR, hazard ratio; 95% CI, 95% confidence interval; NLR, neutrophil to lymphocyte ratio; OS, overall survival; TH group, trastuzumab plus docetaxel group. **Fig. S3.** Forest plots showed independent influences on PFS in the THP group by multivariate analysis. Abbreviations: HR, hazard ratio; 95% CI, 95% confidence interval; NLR, neutrophil to lymphocyte ratio; PFS, progression-free survival; THP group, pertuzumab plus trastuzumab plus docetaxel group. **Fig. S4.** Forest plots showed independent influences on OS in the THP group by multivariate analysis. Abbreviations: HR, hazard ratio; 95% CI, 95% confidence interval; NLR, neutrophil to lymphocyte ratio; OS, overall survival; THP group, pertuzumab plus trastuzumab plus docetaxel group.

## Data Availability

The data of CLEOPATRA that support the findings of this study are available from Vivli, Inc, but restrictions apply to the availability of these data, which were used under license for the current study, and so are not publicly available. Data of real-world research are however available from the authors upon reasonable request and with permission of the Institutional Review Board of Xiangya Hospital.
