# Supplementary material for: Prognostic value of baseline neutrophil/lymphocyte ratio in HER2-positive metastatic breast cancer: exploratory analysis of data from the CLEOPATRA trial
Source: Breast Cancer Res. 2024 Jan 11;26:9. doi: 10.1186/s13058-023-01761-x (PMC10785455; doi:10.1186/s13058-023-01761-x)
Supplement: Supplementary file 1 — Additional file 1: Table S1. Baseline information of patients in the TH group before and after PSM. Table S2. Baseline information of patients in the THP group before and after PSM. Table S3. Characteristics of the TH group patients before and after IPTW. Table S4. Characteristics of the THP group patients before and after IPTW. Table S5. Demographic and disease characteristics of local patients. Table S6. IPTW-adjusted univariate and multivariate analyses of the relationship between OS and clinical factors of TH group. Table S7. IPTW-adjusted univariate and multivariate analyses of the relationship between PFS and clinical factors of THP group. Table S8. IPTW-adjusted univariate and multivariate analyses of the relationship between OS and clinical factors of THP group. Table S9. PSM-adjusted univariate and multivariate analyses of the relationship between PFS or OS and clinical factors of TH group. Table S10. PSM-adjusted univariate and multivariate analyses of the relationship between PFS or OS and clinical factors of THP group. Table S11. Univariate and multivariate analyses of the relationship between PFS and clinical factors of local patients. Fig. S1. After PSM, the KM curves of A PFS and B OS according to the low or high NLR in TH group. After PSM, the KM curves of C PFS and D OS according to the low or high NLR in THP group. Abbreviations: KM, Kaplan-Meier; PSM, propensity score matching; PFS, progression-free survival; OS, overall survival; TH group, trastuzumab plus docetaxel group; THP group, pertuzumab plus trastuzumab plus docetaxel. Fig. S2. Forest plots showed independent influences on OS in the TH group by multivariate analysis. Abbreviations: HR, hazard ratio; 95% CI, 95% confidence interval; NLR, neutrophil to lymphocyte ratio; OS, overall survival; TH group, trastuzumab plus docetaxel group. Fig. S3. Forest plots showed independent influences on PFS in the THP group by multivariate analysis. Abbreviations: HR, hazard ratio; 95% CI, 95% confidence [file 13058_2023_1761_MOESM1_ESM.docx]

Supplementary Table 1

**Baseline information of patients in the TH group before and after PSM**

|  | **TH group** | | |  |  | **TH group after PSM** | | |  |
| --- | --- | --- | --- | --- | --- | --- | --- | --- | --- |
| **Characteristics** | **Low** |  | **High** | **P-value** |  | **Low** |  | **High** | **P-value** |
| **Age, years-no. (%)** |  |  |  |  |  |  |  |  |  |
| <55 | 167(87.0) |  | 153(79.7) | 0.055 |  | 132(89.8) |  | 132(89.8) | 1.000 |
| ≥55 | 25(13.0) |  | 39(20.3) |  |  | 15(10.2) |  | 15 (10.2) |  |
| **Disease type at screening-no. (%)** |  |  |  |  |  |  |  |  |  |
| Nonvisceral | 50(26.0) |  | 36(18.8) | 0.087 |  | 30(20.4) |  | 30(20.4) | 1.000 |
| Visceral | 142(74.0) |  | 156(81.2) |  |  | 117(79.6) |  | 117(79.6) |  |
| **ECOG performance status-no. (%)** |  |  |  |  |  |  |  |  |  |
| 0 | 130(67.7) |  | 101(52.6) | **0.003** |  | 92(62.6) |  | 92(62.6) | 1.000 |
| ≥1 | 62(32.3) |  | 91(47.4) |  |  | 55(37.4) |  | 55(37.4) |  |
| **Hormone-receptor status-no. (%)** |  |  |  |  |  |  |  |  |  |
| Positive | 95(49.5) |  | 90(46.9) | 0.069 |  | 73(49.7) |  | 73(49.7) | 1.000 |
| Negative | 88(45.8) |  | 100(52.1) |  |  | 73(49.7) |  | 73(49.7) |  |
| Unknown | 9(4.7) |  | 2(1.0) |  |  | 1(0.7) |  | 1(0.7) |  |
| **Previous neoadjuvant or adjuvant systemic therapy-no. (%)** |  |  |  |  |  |  |  |  |  |
| No | 99(51.6) |  | 105(54.7) | 0.539 |  | 74(50.3) |  | 74(50.3) | 1.000 |
| Yes | 93(48.4) |  | 87(45.3) |  |  | 73(49.7) |  | 73(49.7) |  |

Abbreviations: PSM, propensity score matching; TH group, trastuzumab plus docetaxel group.

Supplementary Table 2

**Baseline information of patients in the THP group before and after PSM**

|  | **THP group** | | |  |  | **THP group after PSM** | | |  |
| --- | --- | --- | --- | --- | --- | --- | --- | --- | --- |
| **Characteristics** | **Low** |  | **High** | **P-value** |  | **Low** |  | **High** | **P-value** |
| **Age, years-no. (%)** |  |  |  |  |  |  |  |  |  |
| <55 | 111(56.3) |  | 93(46.7) | 0.056 |  | 76(51.4) |  | 76(51.4) | 1.000 |
| ≥55 | 86(43.7) |  | 106(53.3) |  |  | 72(48.6) |  | 72(48.6) |  |
| **Disease type at screening-no. (%)** |  |  |  |  |  |  |  |  |  |
| Nonvisceral | 47(23.9) |  | 36(18.1) | 0.159 |  | 29(19.6) |  | 29(19.6) | 1.000 |
| Visceral | 150(76.1) |  | 163(81.9) |  |  | 119(80.4) |  | 119(80.4) |  |
| **ECOG performance status-no. (%)** |  |  |  |  |  |  |  |  |  |
| 0 | 140(71.1) |  | 131(65.8) | 0.262 |  | 107(72.3) |  | 107(72.3) | 1.000 |
| ≥1 | 57(28.9) |  | 68 (34.2) |  |  | 41(27.7) |  | 41(27.7) |  |
| **Hormone-receptor status-no. (%)** |  |  |  |  |  |  |  |  |  |
| Positive | 97(49.2) |  | 90(45.2) | 0.393 |  | 76(51.4) |  | 76(51.4) | 1.000 |
| Negative | 99(50.3) |  | 109(54.8) |  |  | 72(48.6) |  | 72(48.6) |  |
| Unknown | 1(0.5) |  | 0(0) |  |  | 0(0) |  | 0(0) |  |
| **Previous neoadjuvant or adjuvant systemic therapy-no. (%)** |  |  |  |  |  |  |  |  |  |
| No | 112(56.9) |  | 105(52.8) | 0.414 |  | 78(52.7) |  | 78(52.7) | 1.000 |
| Yes | 85(43.1) |  | 94(47.2) |  |  | 70(47.3) |  | 70(47.3) |  |

Abbreviations: PSM, propensity score matching; THP group, pertuzumab plus trastuzumab plus docetaxel group.

Supplementary Table 3

Characteristics of the TH group patients before and after IPTW

|  | **Unweighted，N(%)** | | |  |  | **Weighted, %** | | |  |  |
| --- | --- | --- | --- | --- | --- | --- | --- | --- | --- | --- |
| **Characteristic** | **Low** |  | **High** | **P-value** | **SMD** | **Low** |  | **High** | **P-value** | **SMD** |
| **Age,years** |  |  |  |  |  |  |  |  |  |  |
| <55 | 167(87.0) |  | 153(79.7) | 0.055 | 0.197 | 84.2 |  | 83.7 | 0.906 | 0.012 |
| ≥55 | 25(13.0) |  | 39(20.3) |  |  | 15.8 |  | 16.3 |  |  |
| **Disease type at screening** |  |  |  |  |  |  |  |  |  |  |
| Nonvisceral | 50(26.0) |  | 36(18.8) | 0.087 | 0.176 | 22.5 |  | 22.9 | 0.934 | 0.009 |
| Visceral | 142(74.0) |  | 156(81.2) |  |  | 77.5 |  | 77.1 |  |  |
| **ECOG performance status** |  |  |  |  |  |  |  |  |  |  |
| 0 | 130(67.7) |  | 101(52.6) | **0.003** | 0.312 | 60.7 |  | 59.8 | 0.853 | 0.020 |
| ≥1 | 62(32.3) |  | 91(47.4) |  |  | 39.3 |  | 40.2 |  |  |
| **Hormone-receptor status** |  |  |  |  |  |  |  |  |  |  |
| Positive | 95(49.5) |  | 90(46.9) | 0.069 | 0.238 | 48.3 |  | 48.2 | 0.977 | 0.023 |
| Negative | 88(45.8) |  | 100(52.1) |  |  | 48.9 |  | 49.3 |  |  |
| Unknown | 9(4.7) |  | 2(1.0) |  |  | 2.8 |  | 2.5 |  |  |
| **Previous neoadjuvant or adjuvant systemic therapy** |  |  |  |  |  |  |  |  |  |  |
| No | 99(51.6) |  | 105(54.7) | 0.539 | 0.063 | 52.8 |  | 52.3 | 0.927 | 0.010 |
| Yes | 93(48.4) |  | 87(45.3) |  |  | 47.2 |  | 47.7 |  |  |

Abbreviations: SMD, standardized mean difference; TH group, trastuzumab plus docetaxel group; IPTW, inverse probability of treatment weighting.

Supplementary Table 4

**Characteristics of the THP group patients before and after IPTW**

|  | **Unweighted，N(%)** | | |  |  | **Weighted****, %** | | |  | |  | |
| --- | --- | --- | --- | --- | --- | --- | --- | --- | --- | --- | --- | --- |
| **Charateristic** | **Low** |  | **High** | **P-value** | **SMD** | **Low** |  | **High** | | **P-value** | **SMD** | |
| **Age,years** |  |  |  |  |  |  |  |  | |  | |  |
| <55 | 111(56.3) |  | 93(46.7) | 0.056 | 0.193 | 51.7 |  | 51.8 | | 0.984 | | 0.002 |
| ≥55 | 86(43.7) |  | 106(53.3) |  |  | 48.3 |  | 48.2 | |  | |  |
| **Disease type at screening** |  |  |  |  |  |  |  |  | |  | |  |
| Nonvisceral | 47(23.9) |  | 36(18.1) | 0.159 | 0.142 | 21.3 |  | 21.3 | | 0.992 | | 0.001 |
| Visceral | 150(76.1) |  | 163(81.9) |  |  | 78.7 |  | 78.7 | |  | |  |
| **ECOG performance status** |  |  |  |  |  |  |  |  | |  | |  |
| 0 | 140(71.1) |  | 131(65.8) | 0.262 | 0.113 | 68.5 |  | 68.6 | | 0.991 | | 0.001 |
| ≥1 | 57(28.9) |  | 68 (34.2) |  |  | 31.5 |  | 31.4 | |  | |  |
| **Hormone-receptor status** |  |  |  |  |  |  |  |  | |  | |  |
| Positive | 97(49.2) |  | 90(45.2) | 0.393 | 0.132 | 47.7 |  | 47.8 | 0.697 | | 0.071 | |
| Negative | 99(50.3) |  | 109(54.8) |  |  | 52.0 |  | 52.2 |  | |  | |
| Unknown | 1(0.5) |  | 0(0) |  |  | 0.3 |  | 0 |  | |  | |
| **Previous neoadjuvant or adjuvant systemic therapy** |  |  |  |  |  |  |  |  |  | |  | |
| No | 112(56.9) |  | 105(52.8) | 0.414 | 0.082 | 54.7 |  | 54.8 | 0.986 | | 0.002 | |
| Yes | 85(43.1) |  | 94(47.2) |  |  | 45.3 |  | 45.2 |  | |  | |

Abbreviations: SMD, standardized mean difference; THP group, pertuzumab plus trastuzumab plus docetaxel group; IPTW, inverse probability of treatment weighting.

Supplementary Table 5

**Demographic and disease characteristics of local patients**

|  | **TH group (n=139)** | | |  |  | **THP group (n=109)** | | |  |
| --- | --- | --- | --- | --- | --- | --- | --- | --- | --- |
| **Characteristic** | **Low** |  | **High** | **P-value** |  | **Low** |  | **High** | **P-value** |
| **Age, years-no. (%)** |  |  |  |  |  |  |  |  |  |
| <55 | 45(67.2) |  | 55(76.4) | 0.206 |  | 38 (69.1) |  | 34 (63.0) | 0.499 |
| ≥55 | 22(32.8) |  | 17(23.6) |  |  | 17 (30.9) |  | 20 (37.0) |  |
| **Disease type at screening-no. (%)** |  |  |  |  |  |  |  |  |  |
| Nonvisceral | 42(62.7) |  | 37(51.4) | 0.179 |  | 29(52.7) |  | 23(42.6) | 0.290 |
| Visceral | 25(37.3) |  | 35(48.6) |  |  | 26 (47.3) |  | 31(57.4) |  |
| **ECOG performance status-no. (%)** |  |  |  |  |  |  |  |  |  |
| 0 | 36(53.7) |  | 45(62.5) | 0.295 |  | 26(47.3) |  | 34(63.0) | 0.100 |
| ≥1 | 31(46.3) |  | 27(37.5) |  |  | 29(52.7) |  | 20(37.0) |  |
| **Hormone-receptor status-no. (%)** |  |  |  |  |  |  |  |  |  |
| Positive | 42(62.7) |  | 61(84.7) | **0.003** |  | 43(78.2) |  | 19(35.2) | **<0.001** |
| Negative | 25(37.3) |  | 11(15.3) |  |  | 12(21.8) |  | 35(64.8) |  |
| **Trastuzumab in**  **neo/adjuvant setting-no.(%)** |  |  |  |  |  |  |  |  |  |
| No | 49 (73.1) |  | 58 (80.6) | 0.299 |  | 35 (63.6) |  | 36 (66.7) | 0.740 |
| Yes | 18 (26.9) |  | 14 (19.4) |  |  | 20 (36.4) |  | 18 (33.3) |  |

Abbreviations: TH group, trastuzumab plus docetaxel group; THP group, pertuzumab plus trastuzumab plus docetaxel.

Supplementary Table 6

**IPTW-adjusted univariate and multivariate analyses of the relationship between OS and clinical factors of TH group**

| **Factor** | **Univariate analysis** | | | **Multivariate analysis** | | |
| --- | --- | --- | --- | --- | --- | --- |
|  | **HR** | **95%CI** | **P-value** | **HR** | **95%CI** | **P-value** |
| Age,years (≥55 vs. <55) | 1.150 | 0.78-1.68 | 0.478 | 1.020 | 0.67-1.54 | 0.933 |
| Disease type at screening (Visceral vs. Nonvisceral) | 2.660 | 1.80-3.93 | **<0.001** | 2.690 | 1.77-4.09 | **<0.001** |
| ECOG performance status (≥1 vs. 0) | 1.430 | 1.08-1.88 | **0.013** | 1.390 | 1.05-1.84 | **0.023** |
| Hormone-receptor status |  |  |  |  |  |  |
| Negative vs. Positive | 1.340 | 1.01-1.77 | **0.044** | 1.390 | 1.04-1.87 | **0.027** |
| Unknown vs. Positive | 1.010 | 0.53-1.93 | 0.970 | 1.480 | 0.74-2.96 | 0.269 |
| Previous neoadjuvant or adjuvant systemic therapy (YES vs. No) | 1.090 | 0.83-1.43 | 0.532 | 1.060 | 0.79-1.41 | 0.700 |
| NLR (High vs. Low) | 1.470 | 1.12-1.94 | **0.006** | 1.600 | 1.21-2.13 | **0.001** |

Abbreviations: HR, hazard ratio; 95% CI, 95% confidence interval; NLR, neutrophil to lymphocyte ratio; IPTW, inverse probability of treatment weighting; TH group, trastuzumab plus docetaxel group; OS, overall survival.

Supplementary Table 7

**IPTW-adjusted univariate and multivariate analyses of the relationship between PFS and clinical factors of THP group**

| **Factor** | **Univariate analysis** | | | **Multivariate analysis** | | |
| --- | --- | --- | --- | --- | --- | --- |
|  | **HR** | **95%CI** | **P-value** | **HR** | **95%CI** | **P-value** |
| Age, years (≥55 vs. <55) | 0.810 | 0.63-1.02 | 0.078 | 0.800 | 0.63-1.02 | 0.069 |
| Disease type at screening (Visceral vs. Nonvisceral) | 1.080 | 0.81-1.46 | 0.594 | 1.120 | 0.83-1.51 | 0.447 |
| ECOG performance status (≥1 vs. 0) | 1.230 | 0.95-1.60 | 0.117 | 1.260 | 0.98-1.63 | 0.073 |
| Hormone-receptor status |  |  |  |  |  |  |
| Negative vs. Positive | 1.110 | 0.88-1.41 | 0.379 | 1.100 | 0.87-1.39 | 0.428 |
| Unknown vs. Positive | 6.010 | 4.54-7.95 | **<0.001** | 7.780 | 4.86-12.46 | **<0.001** |
| Previous neoadjuvant or adjuvant systemic therapy (YES vs. No) | 1.010 | 0.80-1.28 | 0.926 | 0.950 | 0.75-1.20 | 0.650 |
| NLR (High vs. Low) | 1.520 | 1.20-1.93 | **0.001** | 1.540 | 1.21-1.95 | **<0.001** |

Abbreviations: HR, hazard ratio; 95% CI, 95% confidence interval; NLR, neutrophil to lymphocyte ratio; IPTW, inverse probability of treatment weighting; THP group, pertuzumab plus trastuzumab plus docetaxel group; PFS, progress-free survival.

Supplementary Table 8

**IPTW-adjusted univariate and multivariate analyses of the relationship between OS and clinical factors of THP group**

| **Factor** | **Univariate analysis** | | | **Multivariate analysis** | | |
| --- | --- | --- | --- | --- | --- | --- |
|  | **HR** | **95%CI** | **P-value** | **HR** | **95%CI** | **P-value** |
| Age, years (≥55 vs. <55) | 0.970 | 0.71-1.32 | 0.831 | 0.930 | 0.68-1.27 | 0.650 |
| Disease type at screening (Visceral vs. Nonvisceral) | 1.350 | 0.88-2.06 | 0.166 | 1.350 | 0.87-2.08 | 0.177 |
| ECOG performance status (≥1 vs. 0) | 1.720 | 1.25-2.37 | **0.001** | 1.730 | 1.26-2.38 | **0.001** |
| Hormone-receptor status |  |  |  |  |  |  |
| Negative vs. Positive | 0.860 | 0.63-1.18 | 0.349 | 0.830 | 0.61-1.14 | 0.258 |
| Unknown vs. Positive | 8.090 | 5.78-11.31 | **<0.001** | 8.740 | 4.83-15.81 | **<0.001** |
| Previous neoadjuvant or adjuvant systemic therapy (YES vs. No) | 1.200 | 0.88-1.64 | 0.243 | 1.150 | 0.84-1.58 | 0.376 |
| NLR (High vs. Low) | 1.190 | 0.87-1.62 | 0.272 | 1.210 | 0.88-1.65 | 0.237 |

Abbreviations: HR, hazard ratio; 95% CI, 95% confidence interval; NLR, neutrophil to lymphocyte ratio; IPTW, inverse probability of treatment weighting; THP group, pertuzumab plus trastuzumab plus docetaxel group; OS, overall survival.

Supplementary Table 9

**PSM-adjusted univariate and multivariate analyses of the relationship between PFS or OS and clinical factors of TH group**

|  | **PFS** | | | |  | **OS** | | | |
| --- | --- | --- | --- | --- | --- | --- | --- | --- | --- |
| **Factor** | **Univariate** | **Multivariate** | | |  | **Univariate** | **Multivariate** | | |
|  | **p-value** | **HR** | **95%CI** | **P-value** |  | **p-value** | **HR** | **95%CI** | **P-value** |
| Age, years(≥55 vs. <55) | 0.990 | 0.960 | 0.63-1.48 | 0.868 |  | 0.386 | 1.060 | 0.65-1.74 | 0.820 |
| Disease type at screening (Visceral vs. Nonvisceral) | 0.075 | 1.360 | 0.96-1.92 | 0.082 |  | **<0.001** | 3.300 | 1.93-5.63 | **<0.001** |
| ECOG performance status-no. (%) (≥1 vs. 0) | 0.165 | 1.170 | 0.89-1.53 | 0.262 |  | 0.213 | 1.180 | 0.86-1.62 | 0.316 |
| Hormone-receptor status |  |  |  |  |  |  |  |  |  |
| Negative vs. Positive | 0.658 | 0.870 | 0.66-1.13 | 0.293 |  | **0.029** | 0.650 | 0.47-0.89 | **0.007** |
| Unknown vs. Positive | 0.719 | 1.530 | 0.36-6.53 | 0.566 |  | 0.407 | 1.030 | 0.13-8.01 | 0.978 |
| Previous neoadjuvant or adjuvant systemic therapy (Yes vs. No) | 0.571 | 0.910 | 0.70-1.18 | 0.484 |  | 0.695 | 0.900 | 0.66-1.23 | 0.504 |
| NLR (High vs. Low) | **0.030** | 1.350 | 1.04-1.75 | **0.024** |  | **0.007** | 1.610 | 1.17- 2.20 | **0.003** |

Abbreviations: HR, hazard ratio; 95% CI, 95% confidence interval; NLR, neutrophil to lymphocyte ratio; PSM, propensity score matching; TH group, trastuzumab plus docetaxel group; PFS, progress-free survival; OS, overall survival.

Supplementary Table 10

**PSM-adjusted univariate and multivariate analyses of the relationship between PFS or OS and clinical factors of THP group**

|  | **PFS** | | | |  | **OS** | | | |
| --- | --- | --- | --- | --- | --- | --- | --- | --- | --- |
| **Factor** | **Univariate** | **Multivariate** | | |  | **Univariate** | **Multivariate** | | |
|  | **p-value** | **HR** | **95%CI** | **P-value** |  | **p-value** | **HR** | **95%CI** | **P-value** |
| Age, years (≥55 vs. <55) | 0.099 | 0.800 | 0.61-1.06 | 0.127 |  | 0.336 | 0.840 | 0.58-1.21 | 0.349 |
| Disease type at screening (Visceral vs. Nonvisceral) | 0.667 | 1.060 | 0.74-1.52 | 0.757 |  | 0.500 | 1.080 | 0.66-1.76 | 0.770 |
| ECOG performance status-no. (%) (≥1 vs. 0) | 0.114 | 1.280 | 0.93-1.76 | 0.123 |  | **0.004** | 1.730 | 1.17-2.56 | **0.006** |
| Hormone-receptor status |  |  |  |  |  |  |  |  |  |
| Negative vs. Positive | 0.479 | 1.070 | 0.81-1.41 | 0.647 |  | 0.760 | 0.870 | 0.61-1.26 | 0.473 |
| Previous neoadjuvant or adjuvant systemic therapy (Yes vs. No) | 0.364 | 1.020 | 0.77-1.36 | 0.873 |  | 0.121 | 1.200 | 0.82-1.74 | 0.349 |
| NLR (High vs. Low) | **0.003** | 1.520 | 1.15-2.01 | **0.003** |  | 0.494 | 1.130 | 0.79-1.63 | 0.509 |

Abbreviations: HR, hazard ratio; 95% CI, 95% confidence interval; NLR, neutrophil to lymphocyte ratio; PSM, propensity score matching; THP group, pertuzumab plus trastuzumab plus docetaxel group; PFS, progress-free survival; OS, overall survival.

Supplementary Table 11

|  | **TH group (n=139)** | | | | **THP group (n=109)** | | | |
| --- | --- | --- | --- | --- | --- | --- | --- | --- |
| **Factor** | **Univariate** | **Multivariate** | | | **Univariate** | | **Multivariate** | |
|  | **p-value** | **HR** | **95%CI** | **P-value** | **p-value** | **HR** | **95%CI** | **P-value** |
| Age,years(≥55 vs <55) | 0.154 | 2.100 | 1.23-3.59 | **0.006** | 0.072 | 0.590 | 0.33-1.07 | 0.085 |
| Disease type at screening(Visceral vs Nonvisceral) | 0.283 | 1.140 | 0.78-1.67 | 0.484 | 0.437 | 0.790 | 0.51-1.24 | 0.308 |
| ECOG performance s  tatus-no.(%)(≥1 vs 0) | 0.610 | 0.790 | 0.53-1.19 | 0.264 | 0.276 | 1.100 | 0.65-1.88 | 0.713 |
| Hormone-receptor status (Negative vs Positive) | 0.543 | 0.740 | 0.43-1.26 | 0.265 | 0.882 | 0.650 | 0.35-1.18 | 0.154 |
| Trastuzumab in neo/adjuvant setting（Yes vs No) | 0.316 | 1.370 | 0.87-2.15 | 0.176 | 0.740 | 1.220 | 0.78-1.93 | 0.388 |
| NLR (High vs Low) | **0.014** | 1.670 | 1.10-2.53 | **0.015** | **0.032** | 2.040 | 1.20-3.46 | **0.008** |

**Univariate and multivariate analyses of the relationship between PFS and clinical factors of local patients**

Abbreviations: HR, hazard ratio; 95% CI, 95% confidence interval; NLR, neutrophil to lymphocyte ratio; PFS, progress-free survival.


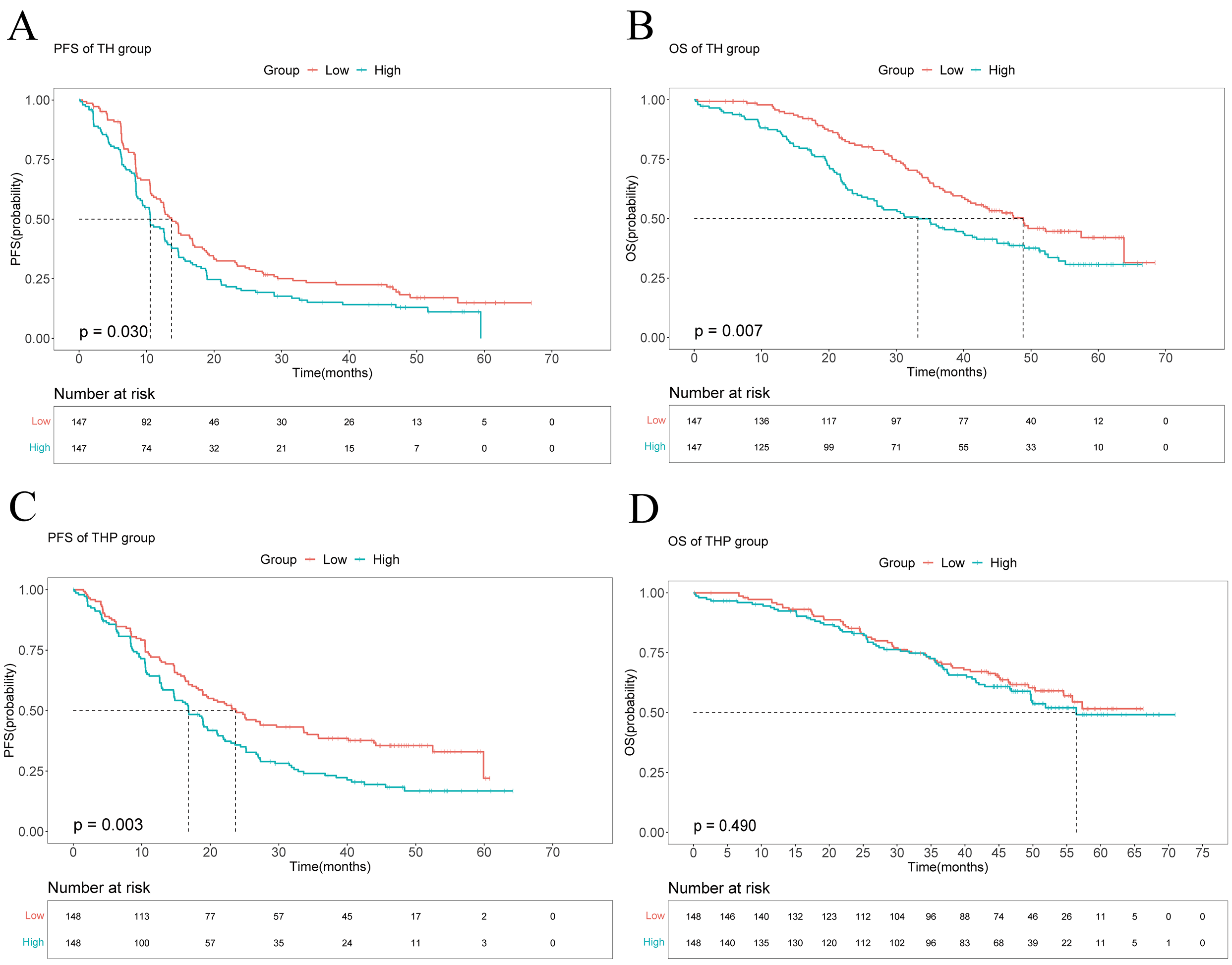


Supplementary Fig1. After PSM, the KM curves of (A) PFS and (B) OS according to the low or high NLR in TH group. After PSM, the KM curves of (C) PFS and (D) OS according to the low or high NLR in THP group.

Abbreviations: KM, Kaplan-Meier; PSM, propensity score matching；PFS, progression-free survival; OS, overall survival; TH group, trastuzumab plus docetaxel group; THP group, pertuzumab plus trastuzumab plus docetaxel.


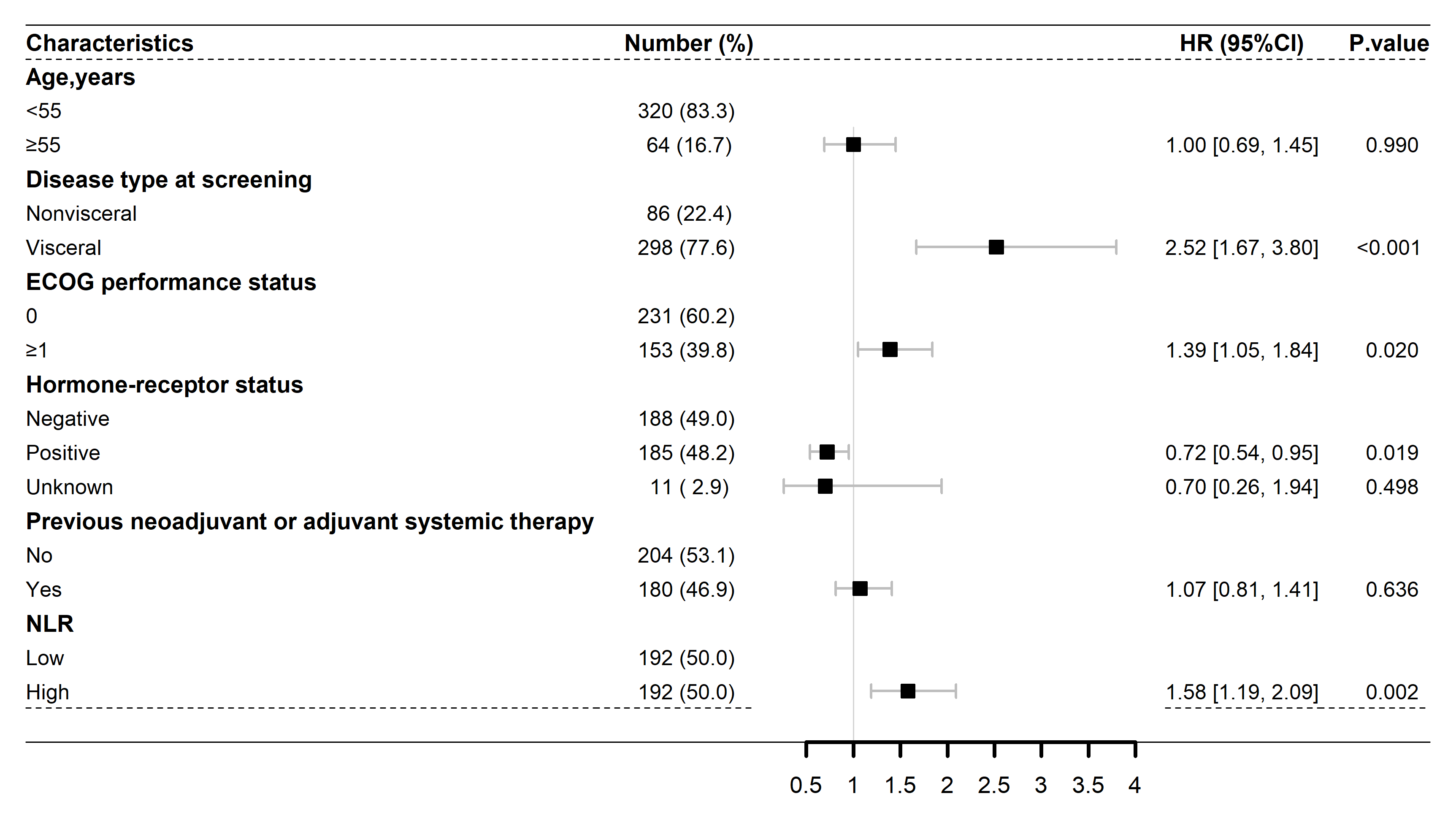


Supplementary Fig2. Forest plots showed independent influences on OS in the TH group by multivariate analysis.

Abbreviations: HR, hazard ratio; 95% CI, 95% confidence interval; NLR, neutrophil to lymphocyte ratio; OS, overall survival; TH group, trastuzumab plus docetaxel group.


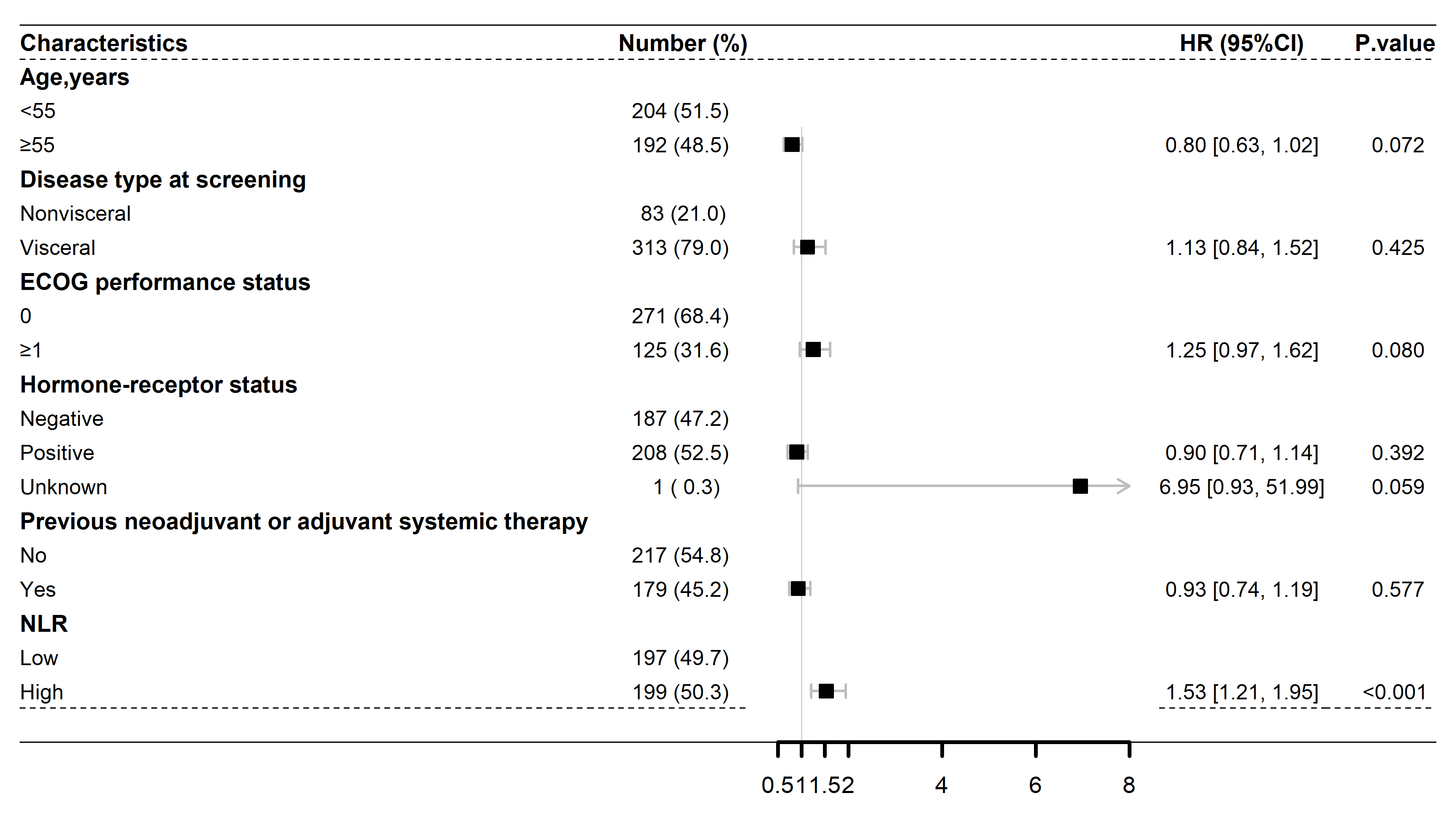


Supplementary Fig3. Forest plots showed independent influences on PFS in the THP group by multivariate analysis.

Abbreviations: HR, hazard ratio; 95% CI, 95% confidence interval; NLR, neutrophil to lymphocyte ratio; PFS, progression-free survival; THP group, pertuzumab plus trastuzumab plus docetaxel group.


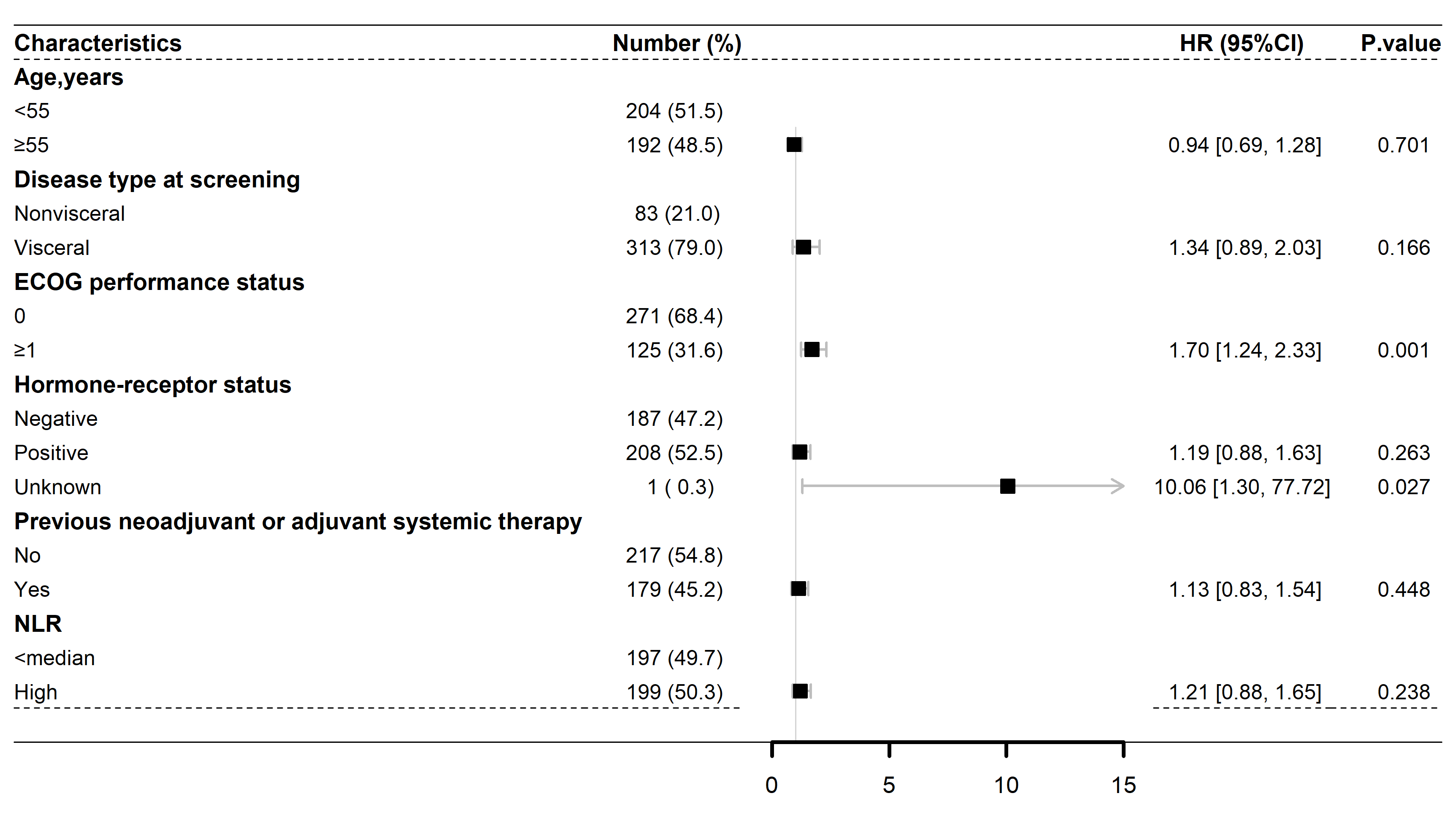


Supplementary Fig4. Forest plots showed independent influences on OS in the THP group by multivariate analysis.

Abbreviations: HR, hazard ratio; 95% CI, 95% confidence interval; NLR, neutrophil to lymphocyte ratio; OS, overall survival; THP group, pertuzumab plus trastuzumab plus docetaxel group.
